# High-throughput proliferation and activation of NK-92MI cell spheroids via a homemade one-step closed bioreactor in pseudostatic cultures for immunocellular therapy

**DOI:** 10.1186/s13036-024-00461-0

**Published:** 2024-11-12

**Authors:** Jhih-Ni Lin, Che-Yung Kuan, Chia-Ting Chang, Zhi-Yu Chen, Wei-Ting Kuo, Jason Lin, Yu-Ying Lin, I.-Hsuan Yang, Feng-Huei Lin

**Affiliations:** 1 Institute of Biomedical Engineering and Nanomedicine, National Health Research Institutes, No. 35, Keyan Road, Zhunan, Miaoli 35053 Taiwan; 2 Department of Biomedical Engineering, College of Medicine and College of Engineering, National Taiwan University, No. 49, Fanglan Rd, Taipei, 10672 Taiwan; 3 National Chung Hsing University, Taichung, Taiwan

**Keywords:** NK cell expansion, Immune cell activation, Custom-made closed bioreactor, Pseudostatic culture, Immunocellular therapy

## Abstract

The NK-92MI cell line has displayed significant promise in clinical trials for cancer treatment. However, challenges persist in obtaining sufficient cell quantities and achieving optimal cytotoxicity. The proliferation of natural killer (NK) cells involves the formation of cell aggregates, but excessively large aggregates can impede nutrient and waste transport, leading to reduced cell survival rates. In this study, a custom bioreactor was designed to mimic pseudostatic culture conditions by integrating brief mechanical rotation during a 6-h static culture period. This method aimed to achieve an optimal aggregate size while improving cell viability. The findings revealed a 144-fold expansion of 3D NK-92MI cell aggregates, reaching an ideal size of 80–150 µm, significantly increasing both cell proliferation and survival rates. After 14 days of culture, the NK-92MI cells maintained their phenotype during the subsequent phase of cell activation. Moreover, these cells presented elevated levels of IFN-γ expression after IL-18 activation, resulting in enhanced NK cell-mediated cytotoxicity against K562 cells. This innovative strategy, which uses a closed suspension-based culture system, presents a promising approach for improving cell expansion and activation techniques in immunocellular therapy.

**Supplementary Information:**

The online version contains supplementary material available at 10.1186/s13036-024-00461-0.

## Introduction

Conventional cancer treatment typically depends on surgery, chemotherapy, and radiation therapy. However, recent progress in cellular immunotherapy has led to the development of innovative methods involving the manipulation of T cells and natural killer (NK) cells to specifically target cancer surface molecules [1]. This emerging technique has garnered significant interest and shows great potential for cancer treatment.

Traditionally, immunocellular therapy utilizing T cells relies on the patient's own immune cells to prevent graft-versus-host disease [2]. Nevertheless, the autologous approach leads to substantial therapeutic expenses and necessitates the expansion of cell production [3]. In contrast, NK cells, which constitute approximately 10% of circulating lymphocytes, have emerged as a safe alternative for therapy [4, 5]. These cells play a crucial role in the rapid cellular immune response by inhibiting the spread of malignant or infected cells. Furthermore, unlike T cells, NK cells do not require prior activation and can promptly initiate spontaneous killing upon encountering malignant cells. Additionally, they can release cytokines that enhance the function and proliferation of other immune cells in the bloodstream and tumor microenvironment [1].

The collection of NK cells typically involves the gathering of peripheral or cord blood, often requiring the use of specific monoclonal antibodies for precise selection [6]. However, there are concerns about the potential effects of these antibodies on NK cell function and the variability in yield, especially when dysfunctional NK cells are sourced from cancer patients. Furthermore, obtaining a sufficient number of NK cells for therapeutic use requires an in vitro expansion process, which is characterized by significant unpredictability due to the complex behavior of lymphocytes under culture conditions [7–9].

An alternative to autologous NK cells is the NK-92 cell line, which originated in the 1990s from mononuclear blood cells taken from patients with aggressive NK-cell lymphoma [10]. The uniformity of the NK-92 cell line offers advantages in terms of engineering feasibility and genetic modification [11, 12]. Additionally, it has shown a favorable safety profile in clinical trials targeting renal, lung, and advanced cancers [13, 14]. While the integration of the NK-92 cell line into various clinical trials has been successful [15–17], a growing body of research suggests that achieving effective results in certain cancer types, such as melanoma and lung cancer, may require higher doses (10^10^ cells) to be not only safe but also necessary for optimal outcomes [13, 14].

Currently, the cultivation of NK cells typically involves static culture methods, often requiring periodic passaging to optimize growth conditions and cell density for faster cell proliferation. In the case of NK-92 cells, their suspension-based growth pattern results in the formation of cellular aggregates [18], which significantly impact cellular behavior, intercellular interactions, and cytokine-mediated signaling within these clusters [19, 20]. In static culture environments, the size of cellular aggregates typically ranges from 200–500 μm [18]. However, larger aggregates may lead to apoptosis in core cells due to limited oxygen and nutrient diffusion, underscoring the importance of achieving an appropriate aggregate size [18, 21]. Nonetheless, studies have shown that NK-92 cells can produce increased quantities of extracellular vesicles through mechanical stimulation in a specialized seesaw-motion bioreactor designed for cancer therapy [22].

**Scheme 1.**
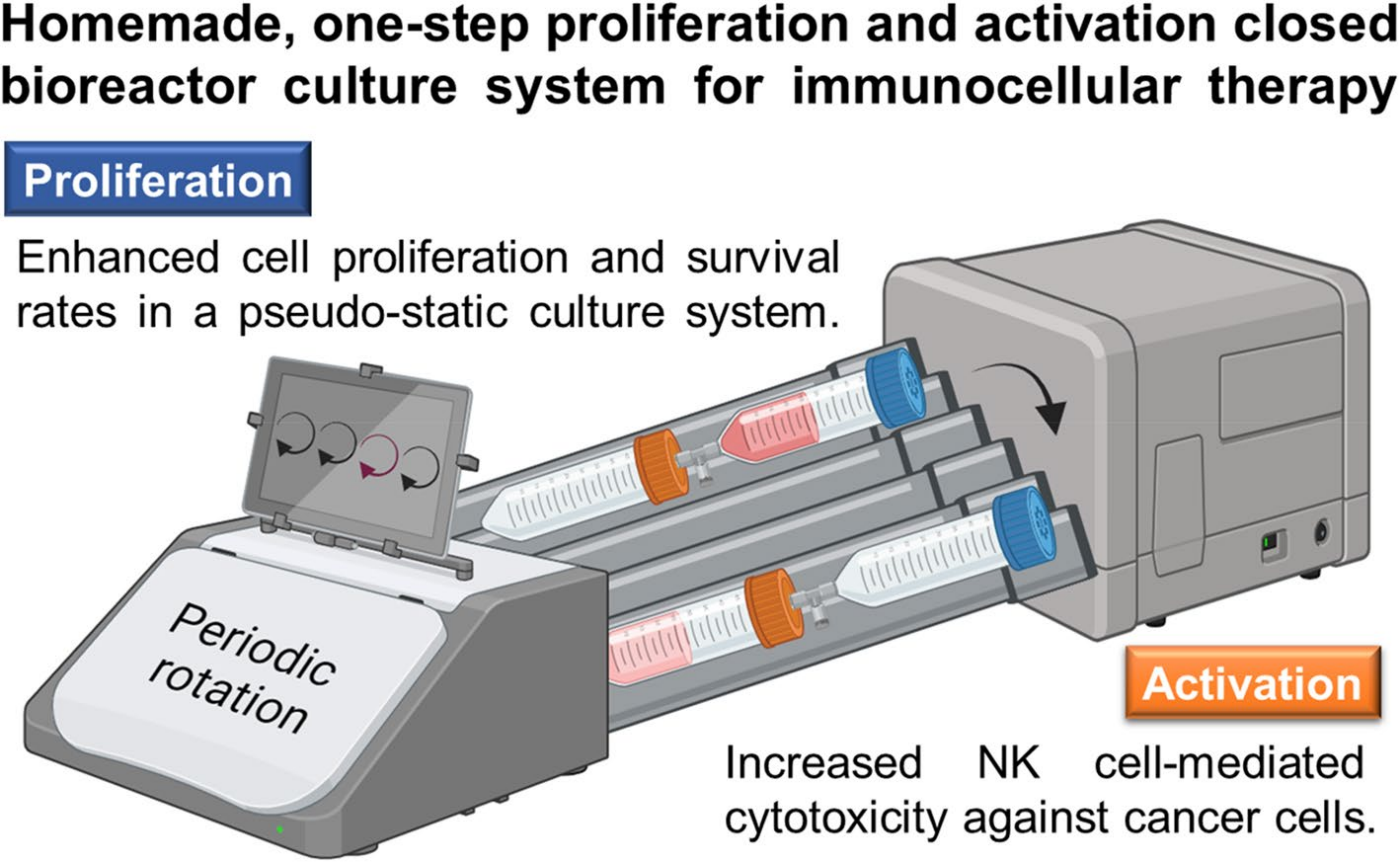
Schematic illustration of a homemade bioreactor that simulates pseudostatic culture conditions for NK-92 MI cell expansion and activation

To address the challenge of cell aggregation and leverage the benefits of dynamic culture environments to increase therapeutic effectiveness, we created a custom bioreactor that mimics pseudostatic culture conditions. This method involves brief mechanical rotation during a 6-h static culture period, with the goal of achieving an optimal cell aggregate size and improving cell viability. This approach eliminates the need for additional passaging, reduces cell loss, and minimizes cultivation disruption. Moreover, we employed NK-92MI cells, a modified IL-2-independent NK-92 cell line known for enhancing immune responses against cancer. To further increase the cytotoxic potential of NK-92MI cells in targeting tumor cells, we introduced a second centrifuge tube into a homemade bioreactor loaded with IL-15 or IL-18. The cells initially multiplied in the upper tube until a specific cell count was reached. Using two-way valves, the proliferated NK-92MI cells were subsequently transferred to the lower tube for activation. We anticipate that NK-92MI cells will be activated, influencing gene expression and demonstrating improved tumor-cell-killing abilities. Compared with conventional NK cell culture methods, the bioreactor system in our study not only addresses apoptosis related to NK cell aggregation but also supports the long-term cultivation and activation of NK cells within a closed system to prevent contamination and ensure user-friendly operation. Furthermore, this approach can be adapted for various immune cells, promoting their growth and activation and offering a high-throughput immune cell culture system for immunocellular therapy. The study design is depicted in Scheme 1.

## Results & discussion

### Proliferation of NK-92MI cells

**Fig. 1:**
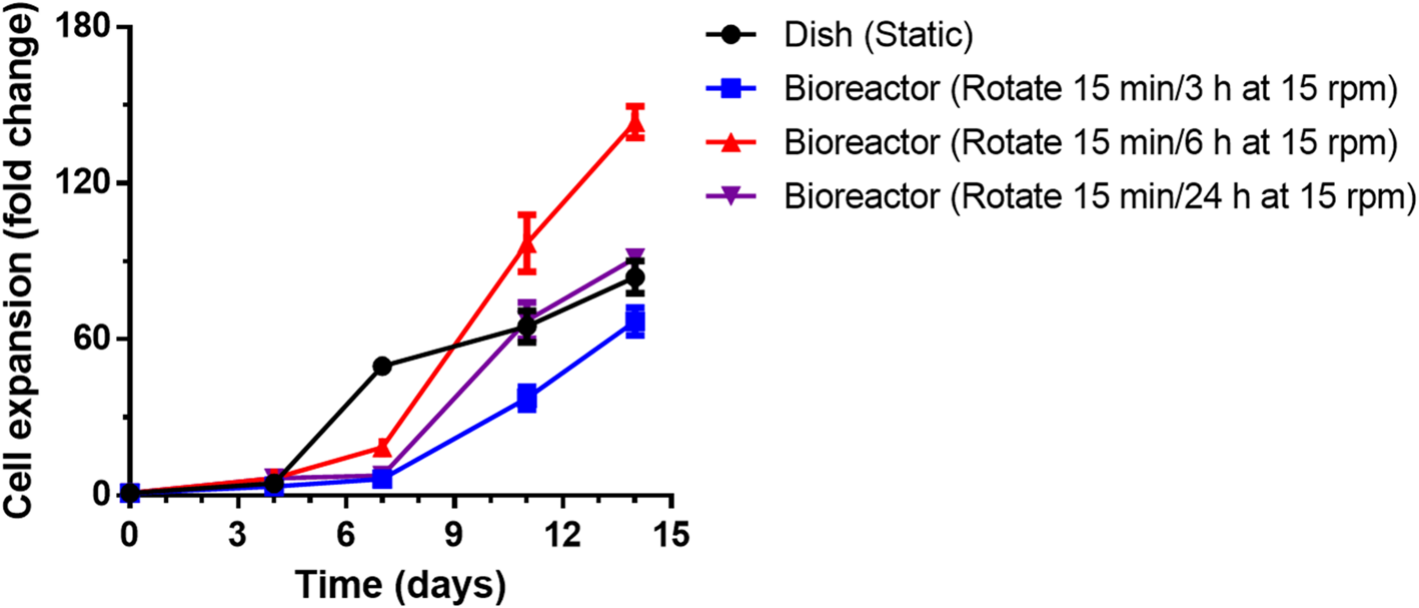
The proliferation of NK-92MI cells cultured in a static dish control system and a pseudostatic bioreactor system on days 4, 7, 11, and 14

To assess the impact of different rotation parameters on overall cell proliferation, various rotation cycles were initially measured (Fig. 1). The cells cultured via traditional methods in static dishes exhibited rapid growth during the initial 7 d. The cells then stabilized and exhibited slow growth, reaching an approximately 84-fold increase in cell number by day 14. This deceleration resulted from the formation of larger cell aggregates, which, in turn, reduced the cell proliferation rate in the later stages of static culture. In contrast, the pseudostatic culture showed relatively slower growth in the first 7 d but grew faster in the subsequent period. This is due to an adaptation phase in the pseudostatic bioreactor during the initial 7 d, during which period rotation assists in optimizing cell aggregation and dispersion. The optimal cell aggregate size significantly affects cell‒cell interactions, increasing the availability of crucial soluble factors, such as growth factors, cytokines, nutrients, and bioactive molecules, which are important for mediating cellular proliferation [23]. In the pseudostatic culture system, an inadequate rotation frequency (15 min/24 h) resulted in suboptimal dispersion, whereas an excessively high frequency (15 min/3 h) affected cell aggregation, consequently impeding proliferation. At an appropriate rotation frequency (15 min/6 h), the cells showed a remarkable 144-fold increase in proliferation by day 14, significantly increasing their potential for cell expansion compared with that of conventional static dish cultures.

Shear stress has also been identified as a critical factor in maintaining cell cultures in bioreactors [24]. The gentle agitation generated by the pseudostatic system's rotation not only promotes uniform distribution of nutrients and oxygen but also applies controlled shear stress to the cells. This helps regulate aggregate size and structure, preventing the formation of overly large clusters that could impede mass transfer. By fine-tuning the rotation parameters, the bioreactor achieves a balance between promoting cell growth and avoiding excessive stress, which could otherwise lead to cell damage or death.

### Viability of NK-92MI cells

**Fig. 2:**
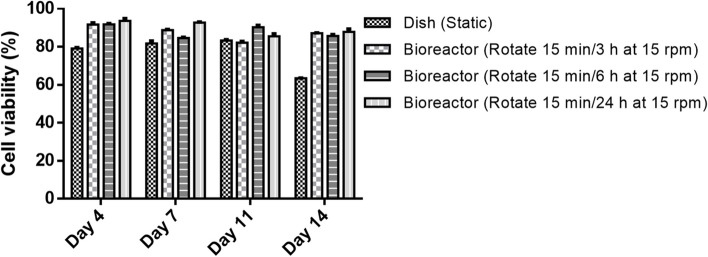
The viability of NK-92MI cells cultured in a static dish control system and a pseudostatic bioreactor system was evaluated via flow cytometry on days 4, 7, 11, and 14

The viability of NK-92MI cells under various culture conditions was assessed by flow cytometry. Although the total cell count was greater after 7 d of static culture (Fig. 1), the increase in cell aggregation and local accumulation of waste slightly reduced cell viability compared with that of the other pseudostatic groups, resulting in approximately 65% viability after 14 d (Fig. 2). In the bioreactor group employing periodic rotation, where the size of the cell aggregates affects the proliferation efficiency, the cell viability consistently surpassed 85% after 14 d. These findings underscore the need for suitable dynamic culture conditions to maintain NK-92MI cell survival. To optimize cell expansion and viability, the parameters were standardized to a rotation of 15 min/6 h at 15 rpm to ensure the most effective cell amplification ratio and sustained cell viability.

### Live/dead cell staining and NK-92MI cell aggregate size

**Fig. 3:**
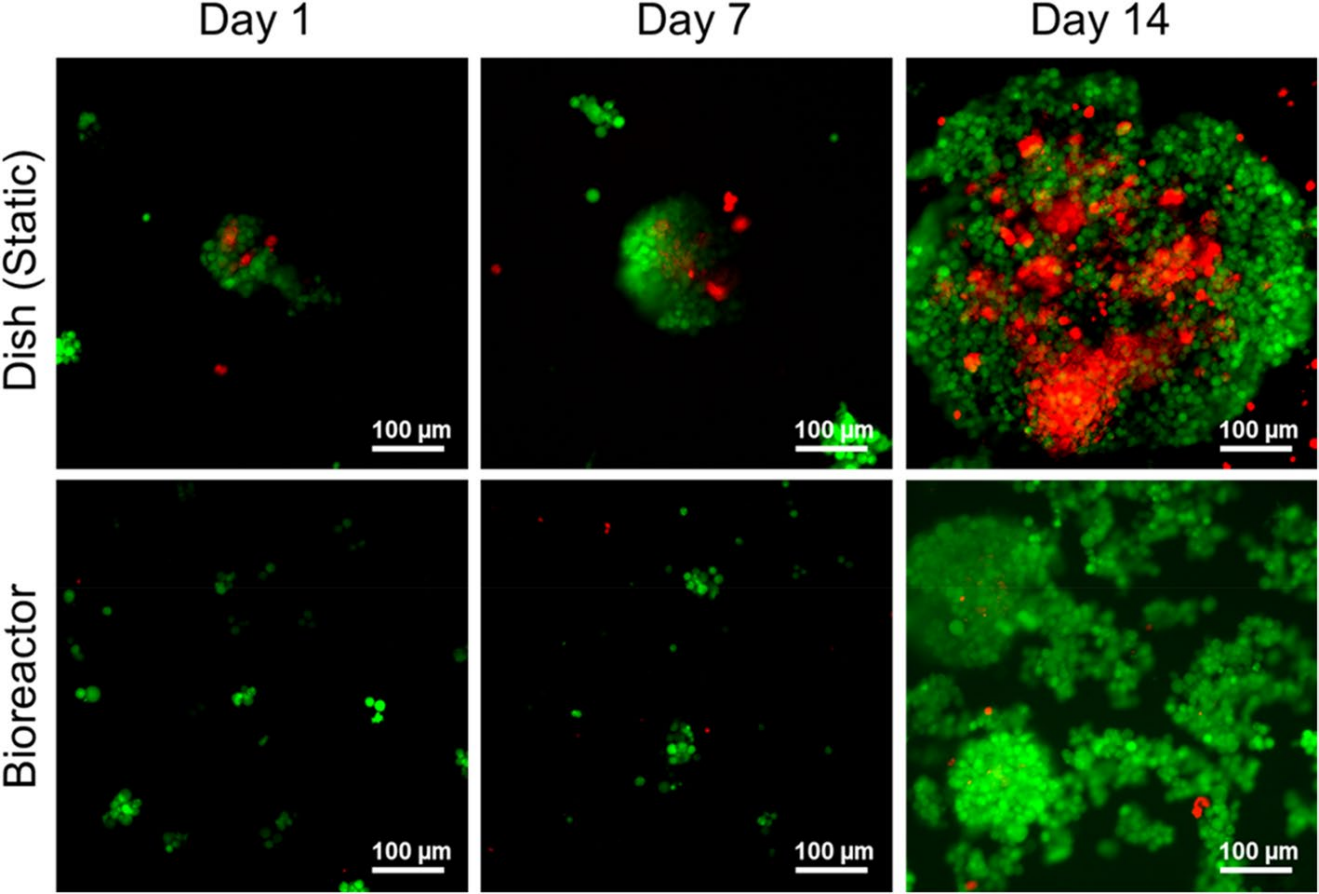
Evaluation of NK-92MI cells via a live/dead cell staining assay after 1–7 and 14 days of cultivation. Living cells in green were stained with calcein AM, and dead cells in red were stained with propidium iodide. Scale bar = 100 μm

After 1, 7, and 14 d of cell culture in both static dishes and bioreactors, meticulously isolated cell aggregates were observed via confocal microscopy to perform live/dead cell staining (Fig. 3). The average aggregate sizes are summarized in Table 1. In static culture, the sizes of the cell aggregates on days 7 and 14 were 140.71 ± 48.97 µm and 395.70 ± 86.15 µm, respectively. Conversely, the periodic shear stress of the bioreactor’s pseudostatic culture notably decreased the cell aggregate size, which averaged approximately 118.44 ± 32.13 µm by day 14. Such variations in aggregate size directly affect survival rates. Although NK-92MI cells cultured in traditional static culture initially grew rapidly (Fig. 1), after 14 days, the inner part of the larger cell mass underwent apoptosis owing to limited mass transfer (Fig. 3). In contrast, the bioreactor group with an appropriate cell aggregate size exhibited fewer instances of apoptosis after 14 days, as depicted in Fig. 2.

**Table 1:** The Average ± standard deviation of cell aggregate size in static dish and pseudo-static bioreactor systems

	**Day 7**	**Day 14**
Dish (Static)	140.71 ± 48.97 µm	395.70 ± 86.15 µm
Bioreactor	66.93 ± 29.20 µm	118.44 ± 32.13 µm

**Fig. 4:**
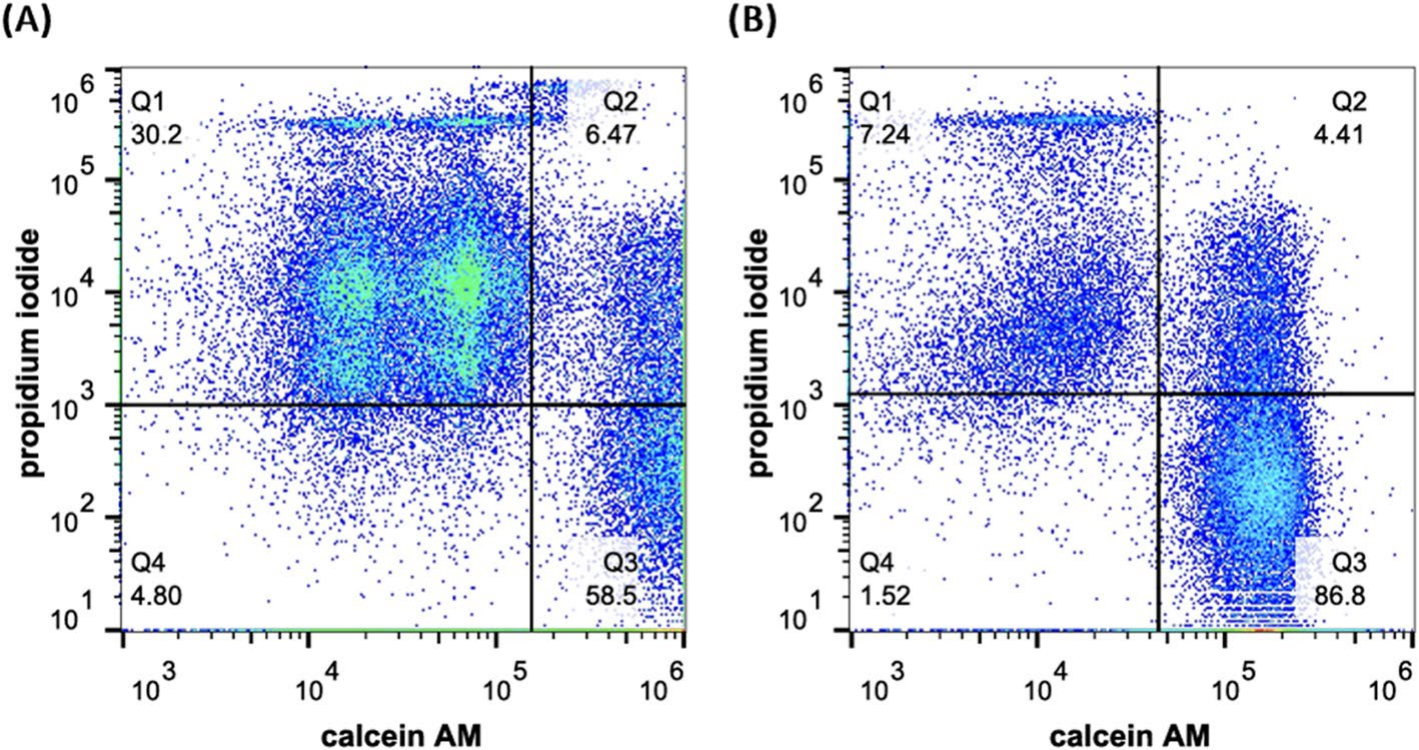
Flow cytometry analysis of the live/dead stained NK-92 MI cells after 14 d of cultivation. **a** Cells were cultured via a traditional static culture method in a culture dish. **b** Cells were cultured via a pseudostatic culture method in a custom pseudostatic bioreactor

**Fig. 5:**
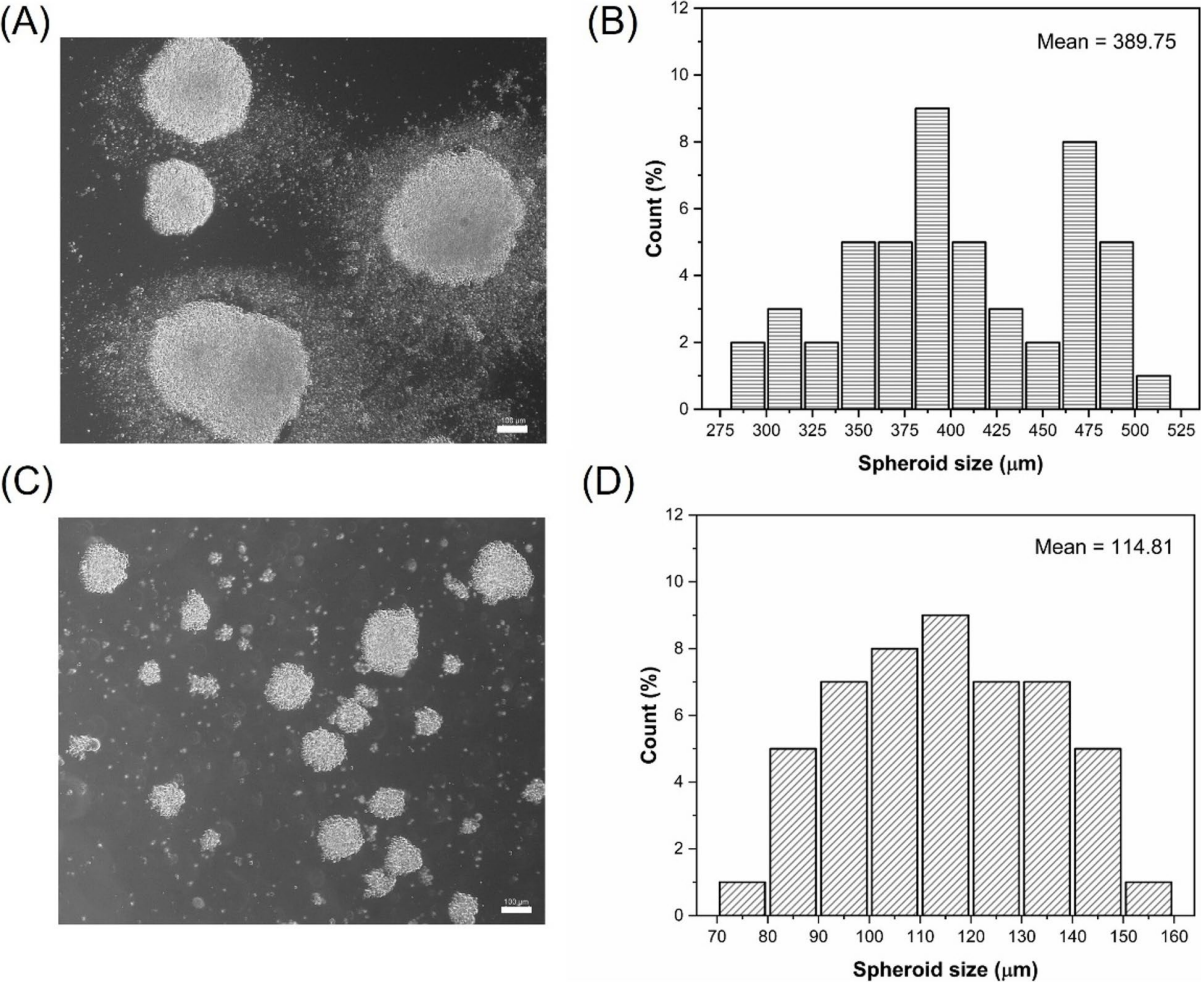
Observation of spheroid morphology and size distribution under static and pseudostatic culture methods after 14 days of cultivation. Bright field images in (**a**) show the morphological characteristics of cell spheroids in static dish cultures, while (**c**) illustrates those in a bioreactor system. The size distributions for the static and pseudostatic culture methods are presented in (**b**) and (**d**), respectively. Scale bar = 100 μm

When the cell aggregate size exceeds 300 µm, nutrient and oxygen transport to and waste transport from the center of the aggregates become suboptimal, leading to apoptosis in the central cells of the aggregates [23–26]. This finding was corroborated by live/dead cell staining after analysis via flow cytometry (Fig. 4). While a certain level of cell death is expected in culture, the data clearly show that the pseudostatic culture method significantly reduces the proportion of dead cells compared to static culture conditions. And Fig. 5 illustrates the general size distribution, indicating that the bioreactor system effectively maintained size control.

These results suggest that the homemade bioreactor system developed in this study effectively generated cell aggregates of appropriate size, promoted cell proliferation and mass transfer, and ultimately improved cell survival rates.

### Phenotypic changes in activated NK-92MI cells in the self-designed bioreactor

**Fig. 6:**
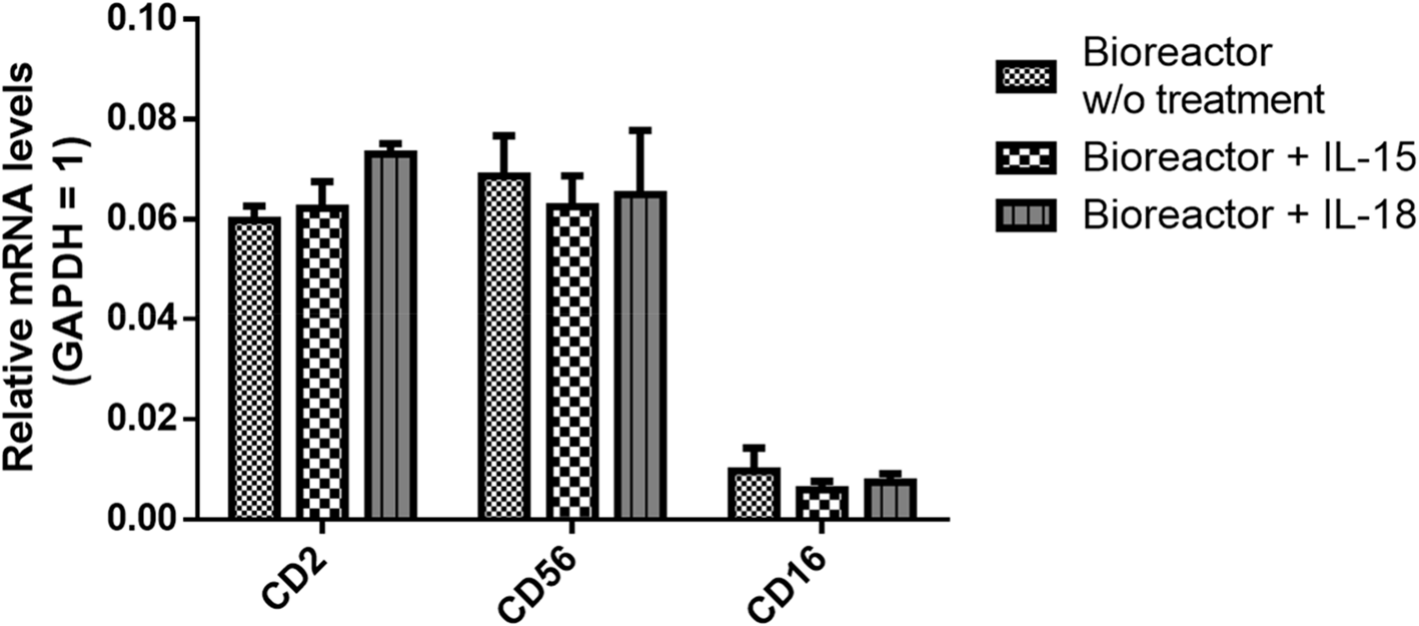
Positive NK-92MI markers, including *CD2* and *CD56*, and the negative NK-92MI marker *CD16* were used to identify the NK-92MI phenotype. The mRNA levels are expressed as relative levels normalized to the *GAPDH* level (defined as 1)

After optimizing the pseudostatic culture of NK-92MI cells, they were further introduced into a self-designed, one-step, homemade bioreactor system, allowing the cells to proliferate within a closed system and undergo IL-15- or IL-18-induced activation. To explore potential alterations in the NK-92MI cell phenotype induced by this culture system, qPCR analysis was performed to measure several NK-92MI cell markers (Fig. 6). The mRNA levels of the positive markers *CD2* and *CD56* were detected in NK-92MI cells, whereas the native negative marker *CD16* was not detected [15, 27]. These findings suggested that neither the bioreactor culture system nor activation with IL-15 or IL-18 significantly altered the specific phenotype of NK-92MI cells.

### Cytotoxicity-related gene expression in NK-92MI cells after homemade bioreactor cultivation

**Fig. 7:**
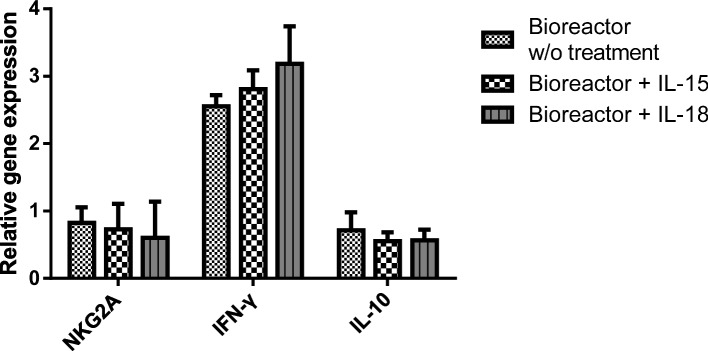
Cytotoxicity-related genes, including *NKG2A*, *IFN-γ*, and *IL-10,* were used to evaluate the increase in gene expression in activated NK-92MI cells. Relative gene expression levels were normalized to *GAPDH* gene expression levels and expressed as the fold change compared with the levels in normal NK-92MI cells

We further investigated the genes associated with immunotherapy and cancer cell killing in NK-92MI cells and compared their expression levels with those of cells cultured conventionally in cell dishes (Fig. 7). NKG2A is an inhibitory NK receptor that recognizes specific ligands on target cells and transmits inhibitory signals [28]. Moreover, NK cells possess the dual capacity to produce proinflammatory (IFN-γ) and anti-inflammatory (IL-10) cytokines, highlighting their essential role both as inflammatory agents for clearing infections and as regulators to manage inflammation and mitigate immune-mediated damage to the host [29, 30]. Higher expression levels of IFN-γ in NK-92MI cells correlate with a stronger ability to kill cancer cells, while IL-10 acts as an antagonist of NK cell activation [31, 32]. Compared with those of normal NK-92MI cells, upon entry into the reactor, the expression levels of genes associated with the inhibition of NK-92MI cell activation, such as *NKG2A* and *IL-10*, were slightly decreased. Conversely, the expression levels of genes related to activated NK-92MI cells, particularly *IFN-γ*, were significantly increased, especially in the IL-15 and IL-18 activation groups. Compared with those in normal NK-92MI cells, the gene expression levels of *IFN-γ* in the IL-15 and IL-18 activation groups were upregulated 2.8- and 3.2-fold, respectively. These findings are consistent with those of previous studies that utilized IL-15 or IL-18 for NK cell activation [32, 33].

### NK-92MI cell-mediated cellular cytotoxicity

**Fig. 8:**
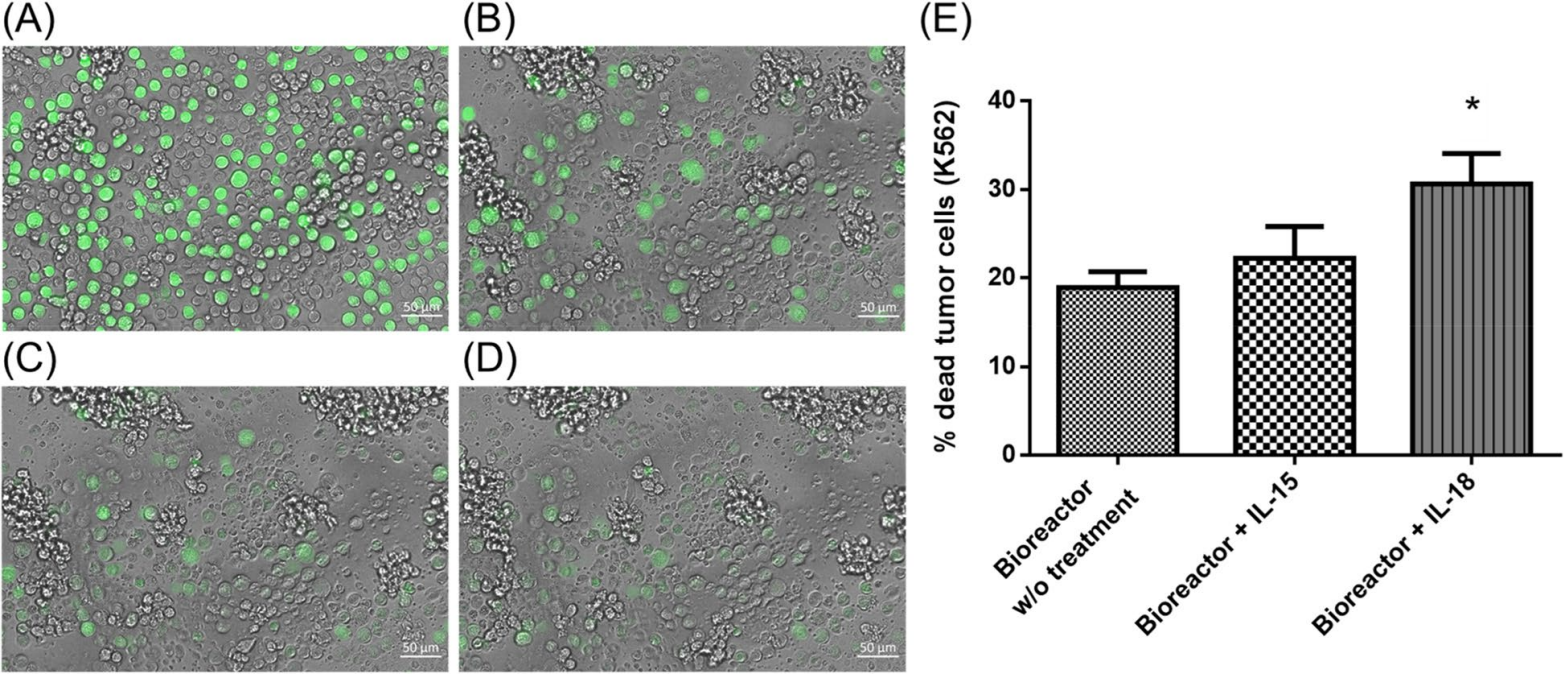
NK-92MI cell-mediated cytotoxicity against K562 cells. After the proliferation and activation of NK-92MI cells induced by IL-18 through a homemade bioreactor, the cells showed a cytotoxic effect on K562 cells after coculture for 0 h (**a**), 2 h (**b**), 3 h (**c**), and 4 h (**d**). **e** Quantification of NK-92MI cell-mediated cytotoxicity was determined by measuring the fluorescence intensity in the supernatant via a plate reader

To determine the activity of NK-92MI cells against cancer cells via the developed homemade one-step proliferation and activation closed bioreactor culture system, the human erythroleukemic cell line K562 was used as the target cell line (Fig. 8).

After labeling with calcein AM, K562 cells exhibited green fluorescence under a fluorescence microscope. After 14 days of proliferation and activation, the NK-92MI cells were cultured. As shown in Fig. 8b–d, within 4 h, the amount of fluorescent dye released from the killed K562 cells gradually decreased, reducing the fluorescence intensity of the cells. The real-time behavior of NK-92MI cells in response to the target K562 cancer cells is shown in Supplementary Video S1. These outcomes enabled further collection of supernatants and the quantitative assessment of cytotoxicity via a specific lysis formula [2, 34]. The results confirmed that the activated cells exhibited enhanced cytotoxicity against K562 cells (Fig. 8e). Specifically, the IL-18-activated group exhibited a 1.62-fold increase in cytotoxicity compared with the nonactivated group. Previous studies have indicated that the cytotoxicity of NK-92MI cells to cancer cells can be increased through IL-18-induced activation [33, 35]. However, IL-15-induced NK-92MI cell-mediated cytotoxicity was not notably pronounced, with a 1.17-fold increase (Fig. 8). It has been reported that IL-15 has some influence on cell survival and can enhance cellular cytotoxicity within a short 24 h period, but this effect diminishes after 96 h [35]. In this study, the relatively modest impact of IL-15 activation on cytotoxicity may be attributed to its suitability for the short-term activation of NK-92 cells.

It has been reported that cytokines captured by NK cells can indirectly trigger signal transduction in surrounding NK cells through cell‒cell interactions within the cell aggregate, maximizing the utilization of cytokines [18, 36]. Therefore, maintenance of aggregate characteristics may be directly related to cell functionality. Additionally, providing adequate mechanical stimulation, including turbulence and stress to NK-92MI cells, would activate NK-92MI cells and generate functional extracellular vesicles of high quality and quantity for cancer treatment [22].

In this study, we designed a custom one-step proliferation and activation closed bioreactor culture system. The NK-92MI cells rapidly proliferated in the upper chamber, forming the adequate cell aggregate size necessary for the desired cell count and survival. Through simple valve control, the 3D NK-92MI cell aggregates were subsequently directed to the lower chamber for specific activation in a single step, avoiding cell loss and cultivation interference during the 14-d culture period. While their distinct cell surface phenotypes are maintained, activated cells enhance the expression of genes associated with cell cytotoxicity, thereby increasing NK-92MI cell-mediated cytotoxicity against tumor cells. This closed system ensures high efficiency, is eco-friendly, is easy to operate, and mitigates the overall experimental contamination risk. Moreover, this design has potential for broader applications in the proliferation and activation of other suspended immune cells, potentially opening new avenues for immunocellular therapy.

## Conclusions

This study details the successful development of a homemade, one-step proliferation and activation closed bioreactor culture system. This system facilitates brief mechanical rotation of 3D NK-92MI cell aggregates during a 6-h static culture period. The expansion of NK-92MI cells reached 144-fold, approximately 1.8 times greater than that in the static control culture system. This approach resulted in the attainment of an optimal cell aggregate size (80–150 µm), leading to improved cell proliferation and survival rates in a pseudostatic culture system. Furthermore, the NK-92MI cells maintained their phenotypes after 14 days of culture and exhibited increased IFN-γ expression levels following IL-18 activation. Notably, the one-step proliferation and activation of NK-92MI cells increased NK cell-mediated cytotoxicity against K562 cells by 1.6-fold compared with that of normal NK-92MI cells. These findings underscore the potential of the homemade functionally closed bioreactor system as a safe and cost-effective tool for immunocellular therapy.

## Materials and methods

### Materials

NK-92MI cells were purchased from ATCC (Manassas, VA, USA). K562 cells were purchased from BCRC (Hsinchu, Taiwan). Minimum essential medium eagle alpha modification (α-MEM), Iscove's modified Dulbecco's medium (IMDM), phosphate-buffered saline (PBS), calcein AM, propidium iodide, and TRIzol reagent were purchased from Thermo Fisher Scientific (Waltham, MA, USA). Inositol and folic acid were purchased from Sigma‒Aldrich (St. Louis, MO, USA). Fetal bovine serum (FBS) and horse serum (HS) were purchased from HyClone (Logan, Utah, USA). The primary antibiotic was obtained from InvivoGen (San Diego, CA, USA). The Direct-zol™ RNA MiniPrep Kit was purchased from Zymo Research (Irvine, CA, USA). The KAPA SYBR® FAST One-Step qRT‒PCR Kit was obtained from KAPA Biosystems (Wilmington, MA, USA).

### Expansion of NK-92 cells

The complete NK culture medium for NK-92MI cells was composed of α-MEM supplemented with 0.2 mM inositol, 0.02 mM folic acid, 12.5% HS, 12.5% FBS, and 0.1% primary antibiotic. NK-92MI cells were cultured in 50 mL centrifuge tubes with a 0.22 μm filter on the cap. Approximately 100,000 NK-92MI cells in 10 mL of complete culture medium were added to a tube and placed on a tube roller (MX-T6-S; DLAB, Beijing, China). The cells were cultured under different pseudostatic culture conditions of brief mechanical rotation at 15 rpm for 15 min after every 3, 6, or 24 h of static culture. They were cultured for 14 d, followed by the addition of 5 mL of medium every 3 d. Cells cultured in a normal Petri dish served as controls.

### Flow cytometry analysis of NK-92 cells

The proliferation and viability of NK-92MI cells cultured in the homemade bioreactor were assessed via flow cytometry on days 1, 4, 7, 11, and 14. First, the cells were collected, washed with PBS and stained with 0.1 μM calcein AM and 3 μM propidium iodide for 15 min at room temperature in the dark. The total and live cells were counted via a flow cytometer (Attune NxT; Invitrogen, Carlsbad, CA, USA).

### Live/dead NK-92MI cell staining

To measure the NK-92MI cell aggregate size and the degree of cell necrosis, the cells were collected on days 1, 7, and 14; washed with PBS; and stained with 2 μM calcein AM and 1 μM propidium iodide for 15 min at room temperature in the dark. The microspheres were observed under a confocal microscope (LSM-900; Zeiss, Oberkochen, Germany). Living and dead cells are shown in green and red, respectively, on the basis of the specific wavelength of light excitation.

### Proliferation and activation of NK-92MI cells in a self-designed bioreactor

NK-92MI cells were cultured in a custom-designed bioreactor consisting of two parts: a 50 mL centrifuge tube with a 0.22 μm filter as the upper tube and a normal 50 mL centrifuge tube as the lower tube. The upper and lower tubes were connected by a two-way valve by drilling a hole with a diameter of 6 mm and were attached via a hot-melt adhesive. Approximately 100,000 NK92-MI cells in 30 mL of complete culture medium were placed in the upper tube, and the lower tube was loaded with 1 mL of 0.9 μg/mL IL-15 or IL-18. The cells were cultured under pseudostatic culture conditions with brief mechanical rotation at 15 rpm for 15 min during a 6-h static culture period for 7 d. On day 8, the cells flowed from the upper tube to the lower tube through the two-way valve and continued culturing under pseudodynamic culture conditions with brief mechanical rotation at 15 rpm for 15 min during a 6-h static culture period for another 7 d.

### Gene expression in NK-92MI cells

On day 14, after the NK-92MI cells were cultured in a custom-designed bioreactor, they were washed with PBS and lysed with TRIzol reagent. The supernatant was collected, and total RNA was extracted via the Direct-zol™ RNA MiniPrep Kit following the manufacturer’s protocol. The RNA samples were analyzed via quantitative polymerase chain reaction (qPCR) via the KAPA SYBR® FAST One-Step qRT‒PCR Kit. The primers used for this analysis are listed in Supplementary Table S1. The intensities were detected and recorded via a ViiA 7 real-time PCR instrument (Applied Biosystems, Foster City, CA, USA). The mRNA levels of *CD2*, *CD16*, and *CD56* were calculated via the comparative Ct method [25], with *GAPDH* used as the reference to calculate relative mRNA levels. Moreover, cytotoxic genes, including *IL-10*, *INF-γ*, and *NKG2A,* were used to evaluate the cytotoxicity of NK-92MI cells. Relative gene expression levels were determined after normalization to *GAPDH* gene expression levels and are expressed as the fold change relative to the control group. NK-92 cells cultured in a normal Petri dish served as controls.

### NK-92MI cell-mediated cytotoxicity

NK cell-mediated cytotoxicity was determined via a calcein AM-labeled K562 cytotoxicity assay. First, K562 cells were stained with 10 µM calcein AM for 30 min and then washed three times with IMDM. Approximately 50,000 NK-92MI cells suspended in 100 μL of complete culture medium obtained from the bioreactor were cocultured with 50,000 calcein AM-labeled K562 cells in 100 μL of IMDM in a round-bottom 96-well plate at 37 °C with 5% CO_2_ for 4 h. Real-time images of NK-92MI cells interacting with K562 tumor cells were captured via the autorecording system of the LSM-900 confocal microscope at a rate of 30 fps. After incubation, the 96-well plate was centrifuged at 120 × *g* for 1 min, and the supernatants (100 μL each) were transferred to a new flat-bottom black 96-well plate for detection. At the end of the reactions, the fluorescence was read in a microplate reader at an excitation wavelength of 488 nm and an emission wavelength of 520 nm. The spontaneously released samples were calcein AM-stained target cells (K562) without effector cells (NK-92MI). The maximum-release samples were stained with Triton X-100. Cytotoxicity was calculated as follows: ${\%\;\mathrm{dead}\;\mathrm{tumor}\;\mathrm{cells}=}\frac{\mathrm{Experimental}\;\mathrm{cell}\;\mathrm{death}\;-\;\mathrm{Spontaneous}\;\mathrm{cell}\;\mathrm{death}\;}{\mathrm{Maximum}\;\mathrm{cell}\;\mathrm{death}\;-\;\mathrm{Spontaneous}\;\mathrm{cell}\;\mathrm{death}}$.

### Statistics

The results obtained in this study are expressed as the means and standard deviations of at least three replicates. One-way analysis of variance with multiple comparisons and Student’s t tests were used for all the statistical analyses. Differences were considered statistically significant at *p* values less than 0.05. (*p *< ​0.05, ∗ ; *p *< ​0.01, ∗ ∗ ; *p *< ​0.001, ∗ ∗ ∗).

## Supplementary Information

Supplementary Material 1.

Supplementary Material 2.

Supplementary Material 3.

## Data Availability

No datasets were generated or analysed during the current study.
