# Supplementary material for: High-throughput proliferation and activation of NK-92MI cell spheroids via a homemade one-step closed bioreactor in pseudostatic cultures for immunocellular therapy
Source: J Biol Eng. 2024 Nov 12;18:65. doi: 10.1186/s13036-024-00461-0 (PMC11555828; doi:10.1186/s13036-024-00461-0)
Supplement: Supplementary file 2 — Supplementary Material 2. [file 13036_2024_461_MOESM2_ESM.pdf]

High-Throughput Proliferation and Activation of NK-92MI Cell Spheroids Using a custom-made One-Step Closed Bioreactor in Pseudo-Static Cultures for Immunocellular Therapy

Jhih-Ni Lin[a,b], Che-Yung Kuan[a,b], Chia-Ting Chang[a,c], Zhi-Yu Chen[a,b], Wei-Ting Kuo[b], Jason Lin[b], Yu-Ying Lin[a,c], I-Hsuan Yang[a,b*], Feng-Huei Lin[a,b,c,*]

Affiliation:

[a] Institute of Biomedical Engineering and Nanomedicine, National Health Research Institutes, No. 35, Keyan Road, Zhunan, Miaoli County 35053, Taiwan.

[b] Department of Biomedical Engineering, College of Medicine and College of Engineering, National Taiwan University, No. 49, Fanglan Rd, Taipei 10672, Taiwan.

[c] PhD Program in Tissue Engineering and Regenerative Medicine, National Chung Hsing University, Taichung, Taiwan.

Corresponding Author

* Feng-Huei Lin: E-mail: double@ntu.edu.tw

* I-Hsuan Yang: E-mail: tony910028@gmail.com

Supplementary Table S1. The primers used for this study.

**Primers**	**Sequence (5' → 3')**
GAPDH forward	ATGGAAATCCCATCACCATCTT
GAPDH reverse	CGCCCCACTTGATTTTGG
CD2 forward	TCAGCTGTCCAGAGAAAGGTC
CD2 reverse	TGCTGGATTCTGAGGGGTTG
CD16 forward	AGACCAGCCCAGATCCAGT
CD16 reverse	TAGCAGAGCAGTTGGGAGGA
CD56 forward	GGCATTTACAAGTGTGTGGTTAC
CD56 reverse	TTGGCGCATTCTTGAACATGA
IL-10 forward	TGCCTTCAGTCAAGTGAAGAC
IL-10 reverse	AAACTCATTCATGGCCTTGTA
INF-γ forward	AAAAATAATGCAGAGCCAAATTG
INF-γ reverse	TAGCTGCTGGCGACAGTTCA
NKG2A forward	GTGATGGCGAAGCGAGTGAAG
NKG2A reverse	CCGAGCCCGAACACACAGAAC
