# Supplementary material for: High-throughput proliferation and activation of NK-92MI cell spheroids via a homemade one-step closed bioreactor in pseudostatic cultures for immunocellular therapy
Source: J Biol Eng. 2024 Nov 12;18:65. doi: 10.1186/s13036-024-00461-0 (PMC11555828; doi:10.1186/s13036-024-00461-0)
Supplement: Supplementary file 3 — Supplementary Material 3. [file 13036_2024_461_MOESM3_ESM.pdf]

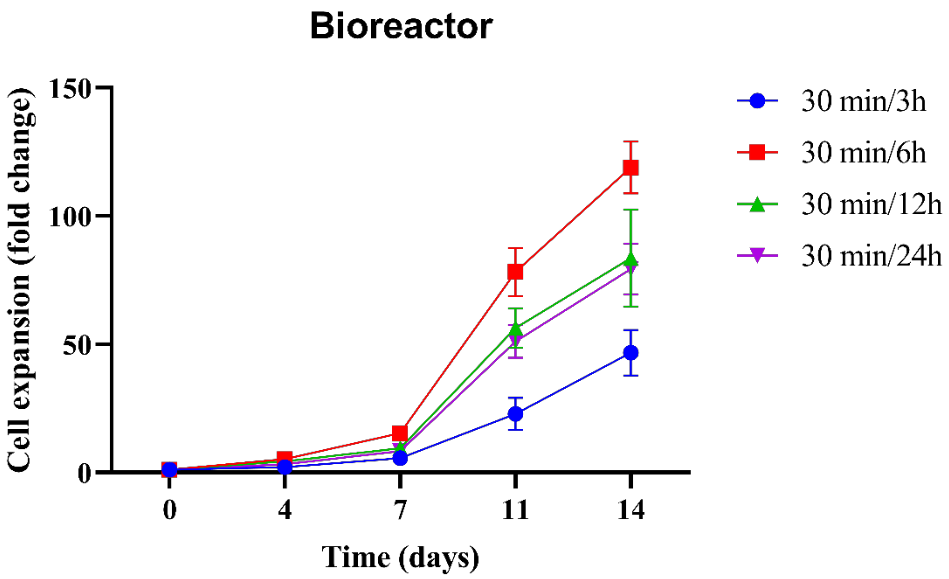

Figure S1. Comparison of cell expansion across different agitation intervals (30 min/3h, 30 min/6h, 30 min/12h, 30 min/24h)
